# Common Changes in Global Gene Expression Induced by RNA Polymerase Inhibitors in *shigella flexneri*

**DOI:** 10.1371/journal.pone.0033240

**Published:** 2012-03-12

**Authors:** Hua Fu, Liguo Liu, Xiaobing Zhang, Yafang Zhu, Lina Zhao, Junping Peng, Hongxuan He, Qi Jin

**Affiliations:** 1 MOH Key Laboratory of Systems Biology of Pathogens, Institute of Pathogen Biology, Chinese Academy of Medical Sciences & Peking Union Medical College, Beijing, People's Republic of China; 2 National Research Center for Wildlife Born Diseases, Institute of Zoology, Chinese Academy of Sciences, Beijing, People's Republic of China; 3 Department of Biological Engineering, College of Life Sciences, Hebei United University, Hebei, People's Republic of China; Louisiana State University and A & M College, United States of America

## Abstract

Characterization of expression profile of organisms in response to antimicrobials provides important information on the potential mechanism of action of the drugs. The special expression signature can be used to predict whether other drugs act on the same target. Here, the common response of *Shigella flexneri* to two inhibitors of RNA polymerase was examined using gene expression profiling. Consistent with similar effects of the two drugs, the gene expression profiles indicated that responses of the bacteria to these drugs were roughly the same, with 225 genes affected commonly. Of them, 88 were induced and 137 were repressed. Real-time PCR was performed for selected genes to verify the microarray results. Analysis of the expression data revealed that more than 30% of the plasmid-encoded genes on the array were up-regulated by the antibiotics including *virF* regulon, other virulence-related genes, and genes responsible for plasmid replication, maintenance, and transfer. In addition, some chromosome-encoded genes involved in virulence and genes acquired from horizontal transfer were also significantly up-regulated. However, the expression of genes encoding the beta-subunit of RNA polymerase was increased moderately. The repressed genes include those that code for products associated with the ribosome, citrate cycle, glycolysis, thiamine biosynthesis, purine metabolism, fructose metabolism, mannose metabolism, and cold shock proteins. This study demonstrates that the two antibiotics induce rapid cessation of RNA synthesis resulting in inhibition of translation components. It also indicates that the production of virulence factors involved in intercellular dissemination, tissue invasion and inflammatory destruction may be enhanced through derepressing horizontal transfer genes by the drugs.

## Introduction

Shigella species are facultative, gram-negative intracellular pathogens responsible for endemic shigellosis, a major worldwide health problem particularly in developing countries. Based on biotyping, shigella is divided into four species: *S. dysenteriae*, *S. flexneri*, *S. boydii*, and *S. sonnei*, of which the disease caused by *S. flexneri* is the most well-understood. The ability of the pathogen to invade and multiply within the gastrointestinal mucosa is essential for Shigella pathogenesis, and structural genes required for invasion and intercellular spreading are encoded within a large virulence plasmid (VP) [1]. Antimicrobial agents are used to control infection; however, the increase in antibiotic resistance of pathogens is threatening to undermine treatment of shigellosis [2].

Two compounds in the rifamycin group of antibiotics, rifampin (RP) and rifaximin (RX), both bind specifically to the beta subunit of the bacterial DNA-dependent RNA polymerase and inhibit RNA synthesis. RP was approved in 1971 to treat individuals with tuberculosis and individuals who are asymptomatic carriers of *Neisseria meningitidis* [3]. RP is also used in combination with other antibiotics to control *Staphylococcus aureus* infection [4]. Alternatively, the U.S. Food and Drug Administration licensed RX in 2004 to treat travelers' diarrhea caused by noninvasive strains of *Escherichia coli*. RX is approved in other countries for the treatment of a variety of gastrointestinal disorders, including acute bacterial infection or colonization by organisms such as *Clostridium difficile*, small bowel intestinal overgrowth, irritable bowel syndrome, inflammatory bowel disease, pouchitis and colonic diverticular disease [5]. RX also effectively prevents shigellosis in healthy volunteers challenged with the *Shigella flexneri* serotype 2a [6]. Importantly, RX possesses an additional pyridoimidazole ring rendering it virtually non-absorbable compared with other rifamycin derivatives. Due to the minimal absorption of RX the risk of adverse effects, systemic toxicity, and drug interactions is correspondingly low compared with systemically available antibiotics.

Transcript profiling using microarray technology allows whole genome level gene expression to be analyzed. Expression profiles of organisms in response to antimicrobials provide important information on the potential mechanism of action of a drug and can determine whether an alternative target exists. In 1999, the first DNA microarray study on bacterial response to antibiotic stress was performed using *Mycobacterium tuberculosis* [7]. The study determined that several genes encoding proteins physiologically relevant to isoniazid's mode of action were induced. Signature genes can be identified using this method because they are regulated as a direct consequence of antimicrobial target inhibition. These genes can be used to predict whether other drugs act on the same target or pathway [8]. However, sometimes signature genes are not specific to a given class of antimicrobials. For example, the target response induced by an RNA synthesis inhibitor can also be triggered by novobiocin, which acts on bacterial DNA gyrase. In addition to identifying signature genes, expressional profiles can also identify genes involved in antibiotic indirect effects, secondary effects and bystander effects [8]. A more reliable method for determining expression signatures unique to a specific class of antibiotics may therefore be to identify distinct combinations of affected genes including the signature genes and other genes related to drug effects.

The transcriptional profile elicited by RP at a minimal inhibitory concentration (MIC) has been reported for *Pasteurella multocida* [9], and the regulation of target genes and a gene locus involved in virulence was observed. However, most of the up-regulated RP-specific genes coded for hypothetical proteins of unknown function. In addition, a study using Salmonella with a promoter-reporter fusion library identified that promoters of some virulence genes were differentially expressed after the addition of sub-MICs of RP [10]. In this study, we investigated the regulation of virulence genes by RNA synthesis inhibitors, RP and RX, in *S. flexneri*, and we defined the common expression signature elicited by the two compounds using whole-genome DNA microarray technology.

## Results

### Antimicrobial activity of RP and RX

**Figure 1:**
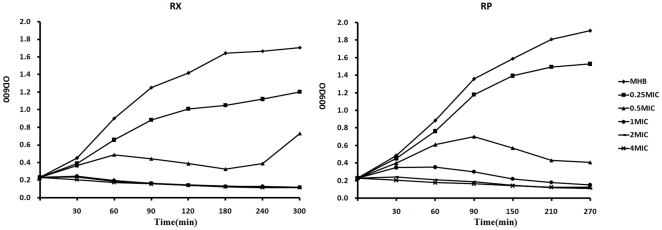
Growth curve for *S. flexneri* in the presence or absence of two RNA polymerase inhibitors.

Both RP and RX exhibit concentration-dependent and time-dependent bactericidal activity on *S. flexneri* (Fig. 1). The growth curve of *S. flexneri* shows that RX suppresses bacterial growth to a higher degree than RP after 90 min of exposure, especially at concentrations no higher than 1×MIC, indicating that the antimicrobial activity of RX is more potent and rapid than that of RP. The two drugs have identical MIC (8 µg/ml); therefore, assessment of antimicrobial activity from MIC alone is not sufficient. The growth rate of *S. flexneri* measured at an optical density of 600 (OD600) was not significantly affected by 0.25×MIC of the drugs. However, growth was inhibited severely by concentrations higher than 1×MIC. To limit secondary effects resulting from growth inhibition, supra-MICs and long incubation periods (more than 60 min) should be avoided. Subsequent microarrays were performed at 0.5×MIC and 1×MIC 10, 30, and 60 min following treatment.

### Overview of transcriptional profiles

**Figure 2:**
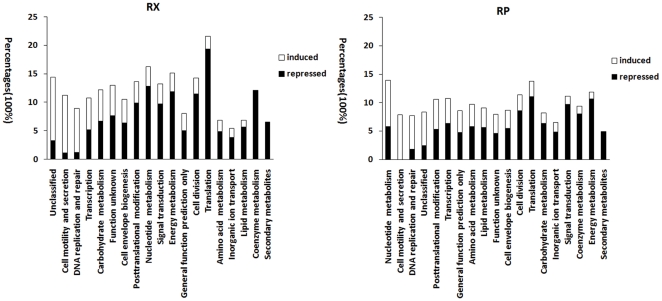
Percentages of genes induced (open bars) and repressed (black bars) for each functional class.

Triplicate data sets were normalized and analyzed as described in the Materials and Methods. Kinetics and concentration dependence of gene expression were examined. The data sets obtained from the present study have been exported to the Gene Expression Omnibus (GEO) in Compliance to MIAME guidelines and can be identified with the accession number GSE 32978. A total of 535 genes had substantially altered expression levels after RX challenge in at least two of the experimental conditions (Table S1). Of these genes, 236 displayed increased expression and 299 displayed reduced expression. To determine the influence of RX on cell biological processes and functions, the differentially expressed genes were categorized using the Clusters of Orthologous Groups of proteins database. A majority of the responsive genes from functional categories including unclassified, cell motility, secretion, DNA replication, DNA repair, and transcription were up-regulated. Most of the responsive genes related to cell metabolism and cell division were down-regulated (Fig. 2).

Compared with RX, a small number of genes (367) were responsive to RP treatment under more than one experimental condition. Specifically, 158 genes were up-regulated and 209 genes were down-regulated (Table S2). Classification of these genes reveals that they are implicated in many aspects of cell function. Expression of RP-responsive genes involved in nucleotide metabolism, cell motility, secretion, DNA replication, and DNA repair was increased. Also induced by RP were many unclassified genes. Alternatively, genes associated with cell functional categories including energy metabolism, translation, and amino acid metabolism were predominately repressed (Fig. 2). These results indicate that the influence of RP on cell function is similar to that of RX, which is consistent with known pharmacological effects of the two compounds. In fact, of the 367 RP-responsive genes, 225 (61.3%) were also identified as RX-responsive genes. These shared genes displayed similar up- or down-regulation trends. However, quantitative differences in transcript levels were observed (Table S3 and S4). To identify indicators of their shared mode of action, we focused our analysis on the genes that displayed a common response to both drugs.

### Commonly induced genes

A striking 42% (37 out of 88 genes) of the shared genes induced by both RP and RX were encoded by *S. flexneri*'*s* large virulence plasmid. These 37 genes make up more than 30% of all plasmid-encoded genes on the array (Table S3). Genes included in this group code for effector proteins secreted by a type III secretion system (TTS), such as *ospG*, *ospB*, *ospF*, and homologs of *ospD* and *ospE*; proteins necessary to assemble TTS such as *mxiA*, *mxiC*, *mxiM*, *spa29*, and *spa33*; virulence factor regulators such as *virF* and *virK*; and other plasmid-linked virulence genes such as s*epA*, *msbB2*, *apy*, *phoN*, *ushA*, and *rfbU*. All of the common responsive genes located on the invasion plasmid were up-regulated, and most of them were expressed in a time-dependent manner, reaching their highest expression after prolonged antibiotic exposure. Some of the plasmid genes also increased in a concentration-dependent fashion.

Also induced by the two drugs in a time-dependent manner were the genes *SF3803* (gene encoding putative secreted autotransporter toxin), *SF0709* (gene encoding putative tail component of prophage CP-933K), *yjhT* (gene encoding N-acetylneuraminic acid mutarotase), *yciF*(gene encoding putative structural protein), *SF1450* (gene encoding putative aldolase), *fimB* and *fimE* (two genes involved in synthesis and expression of type 1 fimbriae), and hypothetical genes *shiA*, *shiC*, *yjiC*, and *yjdA*. The expression patterns of these genes were very similar to those of the plasmid genes, suggesting that they may be cooperatively regulated in response to both drugs.

A distinct set of genes involved in heat shock, including *yrfH*, *hslU*, *htpG*, *clpB*, and *hslO*, exhibited altered levels of gene expression. These genes were most notable at early time point, especially in the test groups treated with high concentrations of the antibiotics. *SF1765*, a putative DNA-binding transcriptional regulator, exhibited a similar expression pattern compared to the heat shock genes. Other common induced genes included *sdaA*, *sdaBC*, *tdk*, *ndk*, *yhdG*, *ybcL*, *mutM*, *ybhE*, *adiY*, *potD*, *ygjO*, *sorE*, *sbp*, *SF2333*, *SF3152*, and *SF1833*. Most of these genes peaked in expression 30 min after treatment at 1×MIC.

### Commonly repressed genes

Expression data for common genes repressed by both compounds are displayed in Table S4. A total of 137 genes were similarly repressed. These genes predominately functioned in normal cell growth and metabolism. They are including genes that code for cold shock proteins, chaperonin GroEL, outer membrane porin protein C, ribosomal proteins, proteins required for citrate cycle, glycolysis, thiamine biosynthesis, purine metabolism, fructose metabolism, and mannose metabolism. The two drugs commonly down-regulated 23 hypothetical genes.

### Validation of microarray data by QRT- PCR

**Figure 3:**
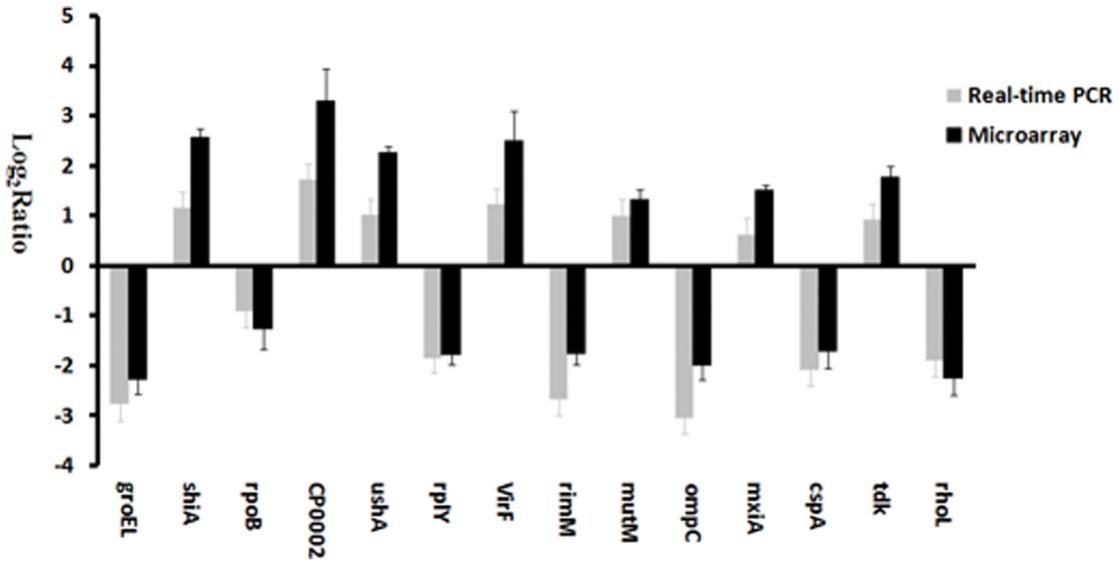
The relative fold change for the genes listed in Table 1 determined by QRT-PCR.

Microarray data were validated with QRT-PCR using the same cDNA preparations used in array hybridizations. A total of 14 genes were selected for validation based on their involvement in a cellular process of interest. A strong positive correlation (Pearson's correlation coefficient, R = 0.97) was observed between the microarray and QRT-PCR data (Fig. 3), indicating that the microarray data are reliable.

## Discussion

In this study, we used whole-genome microarrays to compare changes in *S. flexneri* gene expression induced by treatment with two different antibiotics, RX and RP. The microarrays allowed us to identify common genes responsive to both drugs. Our findings provide insight into the molecular mechanisms of RNA polymerase inhibitors of bacterial growth and further our understanding of the response of bacteria following antibiotic treatment.

RX and RP inhibit the activity of RNA polymerase by binding its beta-subunits encoded by *rpoB* and *rpoC* genes. Previous microarray studies have shown that 1XMIC RP moderately up-regulated the expression of genes related to RNA synthesis [9]. Consistently, we identified that the genes *rpoBC* and *rpoD* (the gene encodingσ70 sigma factor) were induced 1.6-fold to 4.0-fold after incubated with 1XMIC of the two drugs at 10-min time point (Table S3), indicating the inhibition of transcription by them occurred rapidly.

We found that the expression of target genes was concentration-dependent. Therefore, high concentration of the drugs will elicit strong induction. However, the induction of target genes was transient and prolonged incubation with the antibiotics resulted in a rapid decrease in target gene expression (Table S3). Interestingly, after the addition of sub-MICs of both drugs, the decrease in target gene expression over time was halted at the 30-min time point and then began to increase again. As suggested previously [11], we propose that the target genes may be regulated by growth state, which is affected severely by high drug levels, especially after longer periods of treatment. In contrast to the target genes, one gene involved in transcription was continuously inhibited by both drugs. This gene encodes the vital transcription termination protein Rho. *rho* is regulated by Rho-dependent attenuation of transcription [12]. In view of the regulation mechanism, we proposed that the down-regulation of *rho* may be due to the accumulation of free Rho proteins that failed to bind to the nascent messenger RNA at specific Rho termination sites, as a result of the inhibition of the transcription.

Gene expression data revealed that a variety of VP-encoded genes involved in virulence were commonly induced by the two drugs, indicating the regulation of virulence genes is a shared effect for RNA polymerase inhibitors. Compared with previous reports, the number of the virulence genes affected by the drugs in this study was larger. In addition to the VP-encoded genes, some virulence-associated genes located on the chromosome, including *fimB*, *fimE*, *SF3803*, *shiA* and *shiC* were also up-regulated. Among the other chromosome-carried genes showing similar expression pattern with the virulence-associated genes are *yciF*, *SF1450*, *SF0709* and hypothetical genes (*SF2986*, *yjiC* and *yjdA*). We supposed the products of these genes may be related to virulence, although SF0709 encodes putative tail component of prophage CP-933K.

A bacteria's virulence determines its pathogenicity. It is, therefore, necessary to consider the antibiotic's influence on virulence gene expression. A 31-kb region of the *S. flexneri's* VP is essential for entry into epithelial cells. Many virulence-related genes located beyond the 31-kb entry region were significantly altered by the antibiotic. However, with the exception of several components of the Mxi-Spa TTS apparatus, the antibiotics did not influence expression of entry genes and their activator (*virB*). No alteration in the expression of *virA*, a gene outside of the entry region that is regulated by *virB* and involved in invasion and intercellular spreading, was observed. So, in spite of the Osp proteins which play a complementary role to invasion through modulation of host cell signaling pathways were up-regulated, the *virB* regulon and the invasion ability of *S.flexneri* was not activated substantially by the drugs. In addition to TTS system-related genes, several genes carried by the VP are also related to pathogenicity. We found that two proteins, *icsA* and *apy*, essential for intracellular movement and intercellular dissemination [13], [14], and their direct activator (*virF*) were continuously induced by the drugs. The antibiotics also increased the synthesis of a *sepA* encoded secreted serine protease, which is implicated in tissue invasion [15]. The endotoxin of the Shigella LPS is lipid A molecules, which is suggested to play a significant role in aggravating inflammation that eventually destroys the intestinal barrier. We observed enhanced transcription of *msbB2*, which encodes acyl transferases that catalyze the modification of lipid A molecules and contribute to lipid A toxicity [16]. As a result, it is likely that the inhibitors of RNA polymerase contribute to the pathogenesis of shigella upon host cell invasion by enhancing inflammatory destruction of the human epithelium as well as intracellular movement and intercellular dissemination. This presents a risk for application of these antimicrobials to treat shigellosis.

The responsive genes associated with virulence and others which share the similar expression pattern to them comprise a gene cluster in our study. The cluster of genes may be co-regulated in response to a specific signal. Nevertheless, it is difficult to attribute the induction of this cluster of genes to the action of a transcriptional regulator. The function of gene members in this cluster is not confined to virulence. Some VP-encoded genes in the cluster are involved in plasmid replication and maintenance or transfer. Moreover, the distribution of its members is not restricted to the plasmid. Most of the virulence genes exhibited time- and concentration-dependent expression, indicating that the response of the gene is directly attributable to the antibiotic. In shigella it is believed that the VP and the chromosomally encoded SHI-2 island are horizontally acquired [17]. Intriguingly, another gene of the cluster located on the chromosome, *yciF*, was also horizontally acquired in *E.coli* and Salmonella [18]. Therefore, the common feature of this gene cluster may be that they have been acquired by horizontal gene transfer. Rho and H-NS have been implicated in the silencing of horizontally transferred DNA elements, thereby avoiding unintended expression [19]. The expression of the *rho* gene was continuously down-regulated by RX and RP; however, transcription of *hns* did not change. Thus, it is possible that the outbreak of virulence genes and other foreign genes identified in our study may result from reduced *rho* gene expression.

In rapidly growing cells, more than 60% of transcription is allocated towards rRNA, and this is the rate-limiting step in the synthesis of ribosome proteins [20]. As a result, it is not surprising that a number of genes encoding ribosomal components such as ribosome subunits and 16S rRNA-processing protein RimM were repressed by RP and RX. In fact, regulation of ribosomal protein genes was so rapid that they were significantly inhibited only 10 min after the addition of both drugs at 1×MIC. To our knowledge, this rapid decrease in ribosome synthesis is specific to RX and RP. Moreover, a previous study created a compendium of transcriptional profiles in *P. multocida* in reaction to different types of antibiotics and showed that only RNA polymerase inhibitors negatively influenced ribosomal gene transcription [9]. Therefore, the rapid response of the genes related to ribosome components should be regarded as a signature expression change induced by RX and RP.

Yeast cells treated with transcriptional inhibitors have been shown to express heat shock genes [21]. In the present study, a group of heat shock-related genes were induced by RX and RP; however, several cold shock-related genes were down-regulated. Studies have also shown that translation inhibitors can regulate the expression of thermal stress genes [22]. Therefore, the alteration in ribosome synthesis identified in the present study may be involved in controlling expression of thermal stress genes. Nevertheless, the expression pattern of the heat shock genes is similar to that of *rpoB*, *rpoC*, and *rpoD*. Further study is needed to explain this phenomenon.

In conclusion, we obtained a global view of *S. flexneri* gene expression over time in response to two inhibitors of RNA synthesis, RX and RP, using DNA microarray. Microarray data indicate that the drugs induce rapid cessation of RNA synthesis, resulting in rapid inhibition of translation components. Notably, a number of genes acquired by horizontal transfer, particularly virulence genes encoded by VP, were induced by the drugs, possibly through the inhibition of Rho. Some thermal stress genes were differentially regulated by the two drugs. These common expression characteristics as a whole can be used to predict whether a novel agent act on the RNA polymerase. Further study is, however, needed to test if these responsive genes are shared by other inhibitors of RNA synthesis. Our finding suggests processes related to the pathogenesis of shigella including intracellular movement, intercellular dissemination, tissue invasion and inflammatory destruction may be enhanced during antibiotic treatment. These results contribute to a clearer understanding of the regulation of virulence gene expression in *S. flexneri*.

## Materials and Methods

### Bacterial strain, medium and minimal inhibitory concentration determination

*S.flexneri* 2a strain 301(Sf301) was used in our study. The germ was grown at 37°C with shaking (200 rpm) on cation-adjusted Mueller–Hinton broth (caMHB), a medium recommended by Clinical and Laboratory Standards Institute (CLSI) for susceptibility testing. RP and RX purchased from Sigma-Aldrich were resolved in methanol to give a concentration of 51.2 mg ml^−1^ and diluted with caMHB to final concentrations. The minimal inhibitory concentrations (MIC) of the two drugs for Sf301 were determined respectively according to the CLSI broth macrodilution methods for bacteria that grow aerobically, approved standard M7-A7, 7th edn.

### Growth curves

Sf301, taken from a 24 h culture in caMHB, was inoculated in the same medium until reaching an optical density at OD600 of about 0.05. Then, the cultures were allowed to continue growing at 37°C with shaking. When they were grown to early exponential growth phase (OD600 = 0.23), the drugs were added from the 100×stock dissolved in methanol into the cultures to give final concentrations of 0×MIC, 0.25×MIC, 0.5×MIC, 1×MIC, 2×MIC and 4×MIC. The final methanol concentration for all conditions was 1% (v/v). At certain time intervals, bacterial densities were determined by measuring the OD600.

### Drug treatment

When cultures were grown to early exponential growth phase, the drugs were added respectively to give final concentrations of 0.5×MIC and 1×MIC. The final concentration of the solvent (methanol) was 0.25% (v/v). Meanwhile, methanol solution without drug was added to control cultures at the same final concentration. At 10 min, 30 min and 60 min after treatment, samples were collected and washed twice with phosphate- buffered saline at 25°C for subsequent RNA isolation. To prepare biological replicates for RNA isolation, each experiment was independently performed three times.

### RNA isolation and preparation of labeled cDNA

This part of work is performed according to our previous reports [23], [24]. In general, total RNA was extracted and reverse transcribed by using SV RNA Isolation System (Promega) and Superscript II system (Invitrogen) respectively to prepare cDNA. According to the manufacturer's instructions, the cDNA was labeled with incorporation of fluorescent dyes (Amersham). Cy3-dCTP monofunctional dye was selected for control cDNA samples, and for drug-treated samples, Cy5-dCTP was used.

### Microarray construction and hybridizations

The *S. flexneri* whole genome microarrays were designed by using our sequenced strain (Sf301) and were constructed in accordance with previous reports [1], [25]. Generally, Sf301 ORFs were amplified with ORF-specific primer pairs. Amplified products, which mostly ranged between 300 and 700 bases in length, were purified by using MultiScreen-PCR plates (Millipore). Concentrations of PCR products were measured by Spectra MAX 190. Then, the PCR products were readjusted to 100 ng/µl of concentration with deionized water and double diluted into spotting solution containing 60% DMSO. The PCR products were spotted onto gamma amino propylsilan-coated GAPII slides (Corning) by using Omni-Grid™ microarrayer (GeneMachines). The pairs of the labeled cDNA samples were hybridized to the DNA microarrays following the protocol published at http://www.ifr.ac.uk/safety/microarrays/protocols.html#Hybridisations.

### Data acquisition and microarray data analysis

The slides were scanned by using a GenePix 4100A scanner. Fluorescent spots and local background intensities were quantified using GenePix Pro 6.0 software (Axon). Signal intensities were corrected by subtracting the local background value. The data set was filtered so that spots designated as “not found” flagged features; “bad” and “empty” features were excluded from further analysis. Moreover, spots either with signal-to-noise ratio below the detection limit of 3 or containing less than 55% of pixels, which were two standard deviations above background in both channels, were discarded from the data set. Lowess normalizations were performed with the fluorescent signal ratios (Cy5/Cy3) by using MIDAS software (http://www.tigr.org/software/tm4), with a 0.33 smooth parameter. If a gene (spot) was not filtered out during previous analysis in at least two independent experiments and the change of the gene expression was in the same direction, the mean Cy5/Cy3 ratios of the gene were calculated for further analysis. Significant change of expression was identified if the mean ratio of a gene is higher than twofold up- or down-regulated.

### Quantitative real-time PCR (QRT-PCR) assay

Real-time PCR was performed on the ABI 7000 instrument using Power SYBR Green Universal Master Mix (Applied Biosystems). Gene-specific primers (Table 1) were designed using the Primer Premier 5.0 software. Default conditions recommended by the manufacturer were used for real-time PCR. Abundance of each gene was measured relative to a standard transcript, 16S rRNA, and each cDNA was assayed in triplicate PCR reactions.

**Table 1:** Gene-specific primers for quantitative real-time RT-PCR.

Gene	Sense Primer Sequence	Antisense Primer Sequence
*rpoB*	5′ACCTGGTAACTTGCCGTAGCA3′	5′CGGTTGGCGTCATCGTG3′
*mutM*	5′CCATTCTTCATGCGGTGGTG 3′	5′TGGTTGATCGCTTAATCGGTAG 3′
*shiA*	5′TCCGACGCCTTGCTCAAC 3′	5′ACCTGCCGCCAGTCCTTT 3′
*tdk*	5′GCATGGTGCTGCGTCTTGA 3′	5′CGTGGCGATGCCTTTCCT 3′
*virF*	5′AAAGGTGTTCAATGACGGTTAGC3′	5′TGTCAAGGCTTATAATCTCAAATGG3′
*mxiA*	5′TGATAGCGATAATATGGGACGTAAC3′	5′GCCAAGGCAAGAGCTGATGT3′
*CP0002*	5′AATTGTCCACCGTCTGTCAGTAC3′	5′ATACCGTGAACCCTCTGAAAATC3′
*ushA*	5′TTCCAGAATAAAGGCAAAGCAC3′	5′CAGACGTCCGTTCACAACCC3′
*rhoL*	5′ATGCGAAGTGAACAGATTTCTGG 3′	5′CAGAACTGAAACGACAAGACGGA3′
*rplY*	5′CGTAAAGAGCAGGGTAAGGGTG3′	5′CGATGGTTAGAACTTCGCTGTAGA3′
*cspA*	5′ATCACTCCTGACGATGGCTCTA3′	5′GCCGCTTTCGATGGTGAA3′
*rimM*	5′GGGTTCGTCTTACGGTATTCG3′	5′TGTCCTGATTGTGGTGCTTCC3′
*ompC*	5′ACGGCTTCGCAACCTACC3′	5′TCATAAGTAATAGAACCGCCAACG3′
*groEL*	5′TGGACCCAACCAAAGTAACCC3′	5′CATCAGGCCAGCCACAGAA3′

## Supporting Information

Table S1 **Differentially expressed genes of** ***S.flexneri*** **exposed to RX.**
(XLS)

Table S2 **Differentially expressed genes of** ***S.flexneri*** **exposed to RP.**
(XLS)

Table S3 **Expression ratios for target genes and genes that were induced by both RX and RP.**
(XLS)

Table S4 **Expression ratios for genes that were repressed by both RX and RP.**
(XLS)
